# Entrustable Professional Activities (EPAs) for the residency program in vascular surgery

**DOI:** 10.1590/1677-5449.202300752

**Published:** 2023-10-13

**Authors:** Marcos Aurélio Perciano Borges, Ricardo André Viana Barros, Alcides José Araújo Ribeiro, Vanessa Dalva Guimarães Campos

**Affiliations:** 1 Hospital de Base do Distrito Federal - HBDF, Brasília, DF, Brasil.; 2 Escola Superior de Ciências da Saúde - ESCS, Brasília, DF, Brasil.

Medical sciences education is the field in which studies related to health, life, and disease are conducted at postgraduate level. Over the last 20 years, this field has undergone an extremely rapid process of modernization in Brazil. The same process has taken place in parallel in the rest of the Western world, seeking to develop medical education that is more compatible with social reality and founded on the twin pillars of safety and efficacy.^1-4^

According to Ten Cate,^5^ safety is a critical point in medical education, especially so at conclusion of training, when medical activities should be carried out with confidence and free from uncertainty.^5^ In turn, the same author considers that efficacy is directly linked residents’ professionalism and competencies, which originate from their medical experience.^5^

Competencies are a set of characteristics needed to deliver good medical practice and are not limited to acquisition of content and knowledge.^3,4^ The Royal College of Physicians and Surgeons of Canada is a regulator comprising an association of Canadian doctors concerned with defining standards and protocols for competency-based medical education and has gained major acceptance in the global medical community. The College has identified seven essential competencies needed to complete medical residency training, thereby making a counterpoint to and extending the idea of competencies as acquisition of content and knowledge, namely: 1) communication; 2) collaboration; 3) leadership; 4) representativeness in the community; 5) commitment to lifelong learning; and 6) ethical professionalism.^6^

Although reflection on the concept of competence and its educational meaning in medical residency programs is not the central role of this text, it is important to raise an issue related to the term. Commonly, in the field of education, this term has been reconceived as a personal characteristic, trait, or attribute, a behavior or an activity, that draws our attention to extra-personal skills, rather than simply taking into account acquisition of content and knowledge, but also valuing the intrinsic aspects.^2-4^

Several societies of medical specialties in Brazil, such as the Brazilian Federation of Gynecology and Obstetrics Associations (FEBRASGO), have already joined efforts to guide their training and accredited services to consider, validate and use the expansion of these competencies, since the assessment of skills, attitudes and other domains can be achieved in many ways and using reliable protocols.^2^

One current tool that has been widely accepted and disseminated and which deciphers, identifies, and translates the competencies needed to exercise postgraduate medical activity is the concept of Entrustable Professional Activities (EPAs). According to Ten Cate,^5:722^ “EPAs are a new concept in medical education that has aroused much interest among medical educators.” Since their introduction to medical protocols in 2005, the subject has given rise to numerous publications and has been incorporated into competency-based postgraduate programs in many countries. However, few studies have been published in portuguese.^2,4-6^

EPAs are units of competencies and technical guidelines describing specific practical activities that are part of each specialty and are considered by specialists to be indispensable for a professional to be able to act autonomously outside of the educational setting.^3^ They may be delivered by several educators in the same setting to the same learner, can be reproducible as the generations pass, and can be categorized by different levels of complexity, with the constructive objective of fitting in with the pace of learning. However, the degree of commitment of the teaching body is imperative and levels of supervision of residents vary in complexity from the most dependent to the most autonomous and are classified into five levels: 1) the resident just observes the EPA but does not perform it; 2) the resident performs the EPA under direct and proactive supervision; 3) the resident performs the EPA without a supervisor in the room, but readily accessible; 4) the resident performs the EPA with supervision at a distance; and 5) the resident is able to perform the EPA and teach novice residents. These levels are themselves an assessment scale.^5,7^ The insufficient number of educators is a limitation.

Once developed, EPAs must be validated and there are many studies in the literature that present tools for assessment and comparison of groups with results that demonstrate both their efficacy and safety for improving the quality of the professional’s care and the need for improvement, with no global consensus to date on the best means of setting their parameters. One limitation of EPAs is their failure to provide ideal assessment of non-technical skills and behaviors. A systematic review of the literature indexed in specialist databases identified 63 studies on competency-based postgraduate medical education published from 2011 to 2017 and found that the three issues most covered were: 1) there are residents’ learning difficulties; 2) more assertive assessment tools are needed; and 3) it is necessary to seek to improve the performance of the body of preceptors in the application of teachings focused on the necessary skills.^8^

Development of EPAs for the Medical Residency in Vascular Surgery Program could contribute to improved training of specialists, since the concept standardizes competencies, makes them clearer, and affords educators greater reliability with regard to assessment items, making them more objective, fairer, more transparent, and more flexible. EPAs are designed to support residents in acquisition of general and specific skills to be performed individually and autonomously and contribute to bringing the residency program’s educational project up to date.

Development of EPAs must be founded on the matrix of vascular surgery competencies published in Brazil’s Diário Oficial da União on July 7, 2021,^1^ and approved by the National Commission for Medical Residency (Comissão Nacional de Residência Médica - CNRM). The objective of this matrix is to train and certify physicians in vascular surgery, with acquisition of the competencies needed to perform diagnostic procedures, clinical treatment, conventional surgery, and endovascular procedures, for education, research, and delivery of care to patients with congenital, acquired, and degenerative circulatory conditions and in traumatic and non-traumatic emergencies.^1,9^

We believe that the social and humanizing relevance of EPAs means that they will innovate training of vascular surgery specialists, since they are founded on competency-based medical education and take account of quality of care, reduction of risk of medical errors, and improvement of diagnostic resources. They can lead to reduced waste in therapeutic procedures, can increase resolution of problems of differing degrees of complexity, and can improve the fit between professionals’ competencies and their areas of activity in terms of the requirements of hospital demand.^10-16^
