# Supplementary material for: Entrustable Professional Activities (EPAs) for the residency program in vascular surgery
Source: J Vasc Bras. 2023 Oct 13;22:e20230075. doi: 10.1590/1677-5449.202300752 (PMC10706085; doi:10.1590/1677-5449.202300752)
Supplement: Translation

# Translation: Atividades Profissionais Confiáveis (APCs) para os programas de residência em cirurgia vascular

**DOI:** 10.1590/1677-5449.202300751

**Authors:** Marcos Aurélio Perciano Borges, Ricardo André Viana Barros, Alcides José Araújo Ribeiro, Vanessa Dalva Guimarães Campos

**Affiliations:** 1 Hospital de Base do Distrito Federal - HBDF, Brasília, DF, Brasil.; 2 Escola Superior de Ciências da Saúde - ESCS, Brasília, DF, Brasil.

O ensino das ciências médicas, campo que desenvolve estudos relacionados à saúde, à vida e à doença, na pós-graduação, tem passado por um processo de modernização vertiginosa nas últimas duas décadas no Brasil. Paralelamente, esse processo também tem ocorrido no restante do mundo ocidental, buscando encontrar uma formação de ensino mais compatível com a realidade social e alicerçada no binômio segurança-eficiência^1-4^ .

Segundo Ten Cate^5^ , a segurança é um ponto crítico na educação médica, sobretudo na conclusão dos treinamentos, que é uma atividade que deve ser realizada com confiança e sem incertezas^5^ . Já a eficiência, segundo o autor, está diretamente ligada ao profissionalismo e às habilidades do residente, os quais são oriundos da sua experiência como médico^5^ .

As competências são um conjunto de características necessárias para desenvolver uma boa prática médica e não estão ligadas somente à aquisição de conteúdos e conhecimentos^3,4^ . O Royal College of Physicians and Surgeons of Canada, um colégio regulador composto por uma associação de médicos canadenses que se preocupam com o estabelecimento de padrões e protocolos para a educação médica baseada em competências, com grande aceitação na comunidade médica mundial, elenca sete competências necessárias para completar a formação de um médico residente, fazendo, assim, um contraponto e expandindo a ideia de competência como aquisição de conteúdo e conhecimento, a saber: 1) comunicação; 2) colaboração; 3) liderança; 4) representatividade na comunidade; 5) compromisso com o aprendizado contínuo; e 6) profissionalismo ético^6^ .

Embora o conceito de competência e a reflexão sobre o seu significado pedagógico nos programas de residência médica não sejam o papel central deste texto, é importante apontar uma problematização sobre o termo. Comumente, na área da educação, o vocábulo tem sido repensado como uma atribuição, um traço ou uma característica pessoal, um comportamento ou uma ação, que nos chama a atenção para as aptidões extrapessoais, não somente levando em conta a aquisição de conteúdos e conhecimentos, como também valorizando os aspectos intrínsecos^2-4^ .

Diversas sociedades de especialidades médicas no Brasil, como a Federação Brasileira das Associações de Ginecologia e Obstetrícia (FEBRASGO), já reuniram esforços no sentido de orientarem seus serviços formadores e credenciados para considerarem, validarem e utilizarem a expansão dessas competências, já que a avaliação das habilidades, das atitudes e dos demais domínios pode ser realizada de diversas formas e por meio de protocolos confiáveis^2^ .

Uma ferramenta atual que possui grande aceitação e capilaridade, que decifra, identifica e traduz as competências necessárias para o exercício da atividade médica na pós-graduação, são as Atividades Profissionais Confiáveis (APCs), do inglês Entrustable Professional Activities. Segundo Ten Cate^5:722^, as APCs “configuram um novo conceito em educação médica que tem suscitado muito interesse entre os educadores médicos”. Após a sua introdução nos protocolos médicos em 2005, o tema foi fruto de numerosas publicações e foi incorporado nos programas de pós-graduação baseados em competências de diversos países. No entanto, ainda há poucos trabalhos publicados em língua portuguesa^2,4-6^ .

As APCs tratam-se de unidades de diretrizes técnicas e de competências voltadas para determinadas atividades práticas de cada especialidade, consideradas, pelos especialistas, como indispensáveis para um profissional se tornar apto a agir de forma autônoma e fora do ambiente de aprendizagem^3^ . Podem ser aplicadas por vários preceptores, no mesmo cenário, com o mesmo discente, podendo ser reprodutíveis com a passagem das gerações e categorizadas por diferentes níveis de complexidade, com o intuito construtivista de se adequar ao ritmo de aprendizado. Porém, o grau de comprometimento do corpo de preceptores é imperativo, e os níveis de acompanhamento (supervisão) dos(as) médicos(as) residentes podem variar em complexidade, do mais dependente ao mais autônomo, dividindo-se em cinco: 1) o residente não executa, apenas observa a APC; 2) executa-a sob supervisão direta e proativa; 3) executa a APC sem supervisão na sala, mas prontamente alcançável; 4) executa a APC com supervisão a distância; e 5) é capaz de executar e ensinar os residentes iniciantes. Por si só, tais níveis já são um instrumento de avaliação^5,7^ . O número insuficiente de preceptores é uma limitação.

As APCs, após confeccionadas, devem ser validadas, existindo muitos estudos na literatura que demonstram ferramentas de avaliação e de comparação de grupos cujo resultado demonstra tanto sua eficiência e segurança em melhorar a qualidade do atendimento do profissional como a necessidade de aprimoramento, não havendo ainda um consenso mundial sobre a melhor forma de estabelecer seus parâmetros. Como limitações, as APCs não proporcionam a avaliação ideal de habilidades e de comportamentos não técnicos. Numa revisão sistemática da literatura conduzida em bases de dados especializadas, foram elencados 63 estudos sobre educação médica baseada em competências na pós-graduação, entre 2011 e 2017, e neles os três temas mais abordados foram: 1) ainda há dificuldades de aprendizagem dos residentes; 2) necessita-se de ferramentas de avaliação mais assertivas; e 3) é preciso buscar por melhoria do desempenho do corpo de preceptores na aplicação dos ensinamentos voltados para as competências necessárias^8^ .

A elaboração de APCs no Programa de Residência Médica em Cirurgia Vascular poderia colaborar para o aperfeiçoamento dos especialistas, uma vez que tal conceito padroniza as competências, as deixa mais nítidas e dá mais confiabilidade para os preceptores nos pontos de avaliação, tornando-a mais objetiva, justa, transparente e flexível. As APCs procuram auxiliar o pós-graduando na aquisição de habilidades gerais e específicas a serem executadas de forma individual e autônoma, além de colaborar na atualização do projeto pedagógico do programa de residência.

A elaboração das APCs deve estar fundamentada na matriz de competências em cirurgia vascular, publicada no Diário Oficial da União em 07/07/2021^1^ e aprovada pela Comissão Nacional de Residência Médica (CNRM). O objetivo da matriz é formar e habilitar médicos na área de cirurgia vascular para a aquisição de competências necessárias à realização de procedimentos diagnósticos, terapêuticos clínicos, cirúrgicos convencionais e endovasculares, no ensino, na pesquisa e na assistência aos pacientes portadores de afecções circulatórias congênitas, adquiridas, degenerativas, urgências traumáticas e não traumáticas^1,9^ .

Acreditamos que as APCs, dada a sua relevância social e humanizante, inovarão a formação de médicos especialistas em cirurgia vascular, já que estão calcadas numa educação médica baseada em competências e levam em conta a qualidade do atendimento, a redução dos riscos de erros médicos e a melhoria dos recursos usados nos diagnósticos. Nos procedimentos terapêuticos, podem acarretar a redução dos desperdícios, bem como podem incrementar a resolução de problemas em diferentes graus de complexidade e o melhor encaixe das competências dos profissionais e de suas áreas de atuação em relação às necessidades das demandas hospitalares^10-16^ .
